# The important issue of awareness of disaster response to the COVID-19

**DOI:** 10.1016/j.heliyon.2023.e23880

**Published:** 2023-12-20

**Authors:** Sarmini, Faridatul Lailiyah, Suprapto, Mutimmatul Faidah

**Affiliations:** Universitas Negeri Surabaya, Indonesia

**Keywords:** Disaster response awareness, Large-Scale Social Restrictions (LSSR), Covid-19 pandemic, Social construction

## Abstract

This study aimed to analyze the community's disaster response awareness during the Covid-19 pandemic during the implementation of The Large-Scale Social Restrictions (LSSR) in Gresik. Self-awareness was observed using Peter L. Berger and Thomas Luck man's Social Construction Theory through a dialectical process of internalization, objectification, and externalization. The results showed that there had been no good awareness in efforts to prevent the spread of Covid-19 in Gresik. Socially, the community had not taken the dangers of Covid-19 seriously. There was also an inconsistency between knowledge and reality related to disaster response. In coping with the dialectical process, the community had not implemented a disaster-aware culture by obeying the existing regulations. At least, the sociocultural environment determined a person's construction for self-identification, interaction, and adjustment to the social changes that occurred. Hence, the sociocultural construction of the community had never made the disease outbreak a serious problem and considered it as well as God's reminder even though infected cases continued to increase. A situation was indeed difficult for the Task Force to succeed in the Large-Scale Social Restrictions (LSSR) to Community Activities Restrictions Enforcement (CARE) suppressed the increase of Covid-19 positive cases in Gresik, Indonesia. This research sees that the government's policies in handling Covid- 19 are not enough, it needs more optimal involvement of community and religious leaders, to provide education on the importance of maintaining health protocols and building self-awareness of the dangers of Covid.

## 1 Introduction

Corona Virus Disease (Covid-19) has spread throughout the world rapidly starting from the end of December 2019. It has infected millions of people around the world and can cause death. Therefore, this problem is designated as a global pandemic by World Health Organization [[1], [2], [3], [4], [5]]. Such problematic condition is included in the category of non-natural disasters with the category of disease outbreaks. Therefore, knowledge of disaster management is needed to reduce the possibly hazardous impact of the disaster by immediately reacting during and after the disaster along with taking steps to recover from it [6]. Knowledge of disaster management plays an important role by ensuring the availability and access to disaster so that more accurate and reliable information is available.

Disaster management is an essential knowledge given to the community [7]. Therefore, it is important to embrace knowledge, access, use, and distribution of decision quality as a form of disaster response [8]. The involvement of various sectors through public coordination is important to reduce risks after disasters [9]. This includes the roles and responsibilities of national and local governments in collaboration with relevant stakeholders including public and private sectors [10].

In fact, disaster management is multi-sectorial, multi-stakeholder, and multi-hazard, so that the key to success is good coordination and command [11]. However, sometimes coordination has complex problems and is not easy to solve individually. Consequently, partnerships and collaborations must be taken into account in conducting disaster management [12]. Good disaster management is not only in emergency phase but also before and after disaster occurred [13]. Since 2007, Indonesia has implemented a law to regulate disaster management covering disaster preparedness, early warning, and disaster mitigation [14].

Disaster preparedness is undertaken to minimize the adverse effects of hazards through effective prevention, rehabilitation, and recovery measures to ensure timely and effective arrangement and delivery of aid and assistance after a disaster [15]. In a sociological perspective, disaster is often understood based on people's perceptions and are closely related to emotional experiences when their survival is threatened. Therefore, it is categorized as part of socio-cultural context of community experiencing threats [16].

On March 15, 2021, additional cases were still increasing amounted of 120,399,298 confirmed positive cases, 2,664,622 deaths, and 96,944,566 recovered cases. The United States still became the country with the highest number of cases in the world, with 30,080,223 confirmed cases, 547,191 deaths, and 22,168,542 recovered cases [17]. Table 1 portrays 10 countries with the highest number of Covid-19 infection cases as of March 15, 2021.

**Table 1:** Covid-19 cases in the world.

No	Country	Covid-19 Cases			
		Infected	Died	Recovered	
1.	United States of America	30.080.223	547.191	22.168.542	
2.	Brazil	11.483.370	278.229	10.063.808	
3.	India	11.385.158	158.762	11.005.445	
4.	Russia	4.390.608	92.090	3.995.309	
5.	United Kingdom	4.258.438	125.516	3.496.925	
6.	France	4.071.662	90.429	272.960	
7.	Italy	3.223.142	102.145	2.589.731	
8.	Spain	3.183.704	72.258	2.857.714	
9.	Turkey	2.879.390	29.489	2.701.076	
10.	Germany	2.578.835	73.959	2.358.000	

In regard to Table 1, public health control measures to limit the global spread of the virus must be implemented [18]. It is important to limit human-to-human transmission in order to reduce secondary infections among nearby people and medical personnel and to prevent stronger transmission and international spread [19]. Based on the previous management of MERS and SARS, WHO recommends reducing the risk of transmission of acute respiratory infections by avoiding close contact with people suffering from acute respiratory infections, washing hands frequently after direct contact with infected people or environment, and avoiding direct contact with livestock and wild animal. In addition, patients with symptoms of acute respiratory infections must have cough etiquette by maintaining distance, coughing and sneezing using a tissue or cloth, and benefitting health facilities (i.e. hospital [20].

Experience from the early phase of SARS-Cov-2 pneumonia greatly highlights the early detection and isolation of SARS-Cov-2 pneumonia cases [21]. These steps are taken in an effort to identify cases and contacts occurring in the United States as appropriate assessment and treatment of travelers arriving from China mainland to America [22]. Various policies have been implemented by the affected countries such as the implementation of Lockdown, limiting public transportation, canceling social activities, staying at home and maintaining distance, delaying school activities, using delivery services, and implementing clean and healthy lifestyle [23].

Until March 14, 2021, Indonesia was still in the process of fighting the disease outbreak and there had been 1,450,132 confirmed cases out with 131,828 active cases, 39,339 deaths from 59,564 suspects, and 1,278,965 recovered cases from 73,460 specimens [24]. Table 2 shows the ten provinces with the number of Covid-19 cases by March 14, 2021.

**Table 2:** Covid-19 cases in Indonesia.

No	Province	Covid-19 Cases			
		Infected	Died	Recovered	Treated
1.	DKI Jakarta	359.987	5.784	336.771	17.432
2.	West Java	231.692	2.570	180.959	48.163
3.	Central Java	160.896	6.333	107.107	47.456
4.	East java	134.595	8.596	110.992	15.007
5.	East Kalimantan	59.850	1.362	49.355	9.133
6.	South Sulawesi	58.315	847	52.927	4.514
7.	Bali	36.748	576	13.305	22.867
8.	Riau	32.723	783	30.038	1.902
9.	Banten	32.140	527	22.001	9.612
10.	West Sumatera	30.203	657	27.748	1.798
	Total	1.137.149	28.035	931.203	177.884

At East Java Province, Gresik is in the sixth place with total cases of 5311 confirmed positive cases, 69 cases hospitalized, 4891 cases recovered, and 351 cases died [25]. The data contribute to the spread of Covid-19 in Gresik, namely adding 4 confirmed positive cases from the previous total of 5,307, adding 1 death case from the previous total of 350, and adding 9 recovered cases from the previous total of 4882 as of March 19, 2020. So, there are additional confirmed positive, died, and recovered cases within a day in Gresik [26].

Various efforts to suppress the spread of the virus are still being carried out from all community levels because the virus is still contributing to massive destruction [27]. One of them is the implementation of Large-Scale Social Restrictions (LSSR) in accordance with the Regulation of the Minister of Health of the Republic of Indonesia Number 9 of 2020 as an implementation of Law Number 6 of 2018 concerning Health Quarantine, namely in article 2 paragraph 1, article 3, and article 4 paragraph 1, 2.3 [28] and the issuance of the Decree of the Chief of Police Number: Mak/2/III/2020 concerning Compliance with Government Policies in Handling the Spread of the Corona Virus (Covid-19).

Gresik Regency carried out various efforts to prevent the spread of Covid-19 including LSSR and implementing Community Activities Restrictions Enforcement (CARE) as evidenced by the issuance of a letter numbered 360/12/437.96/2021 regarding the implementation of the Java Bali CARE on January 9, 2021 and Circular Letter Number 1 of 2021 concerning the Enforcement of Restrictions on Community Activities to Control the Spread of Covid-19 19 in Gresik on January 10, 2021 in accordance with the Presidential Instruction of the Republic of Indonesia Number 6 of 2020 concerning Increasing Discipline in Enforcement of Health Protocol Laws in the Prevention and Control of Covid-19 during the Implementation of Community Activity Restrictions Enforcement (CARE) [26].

Based on this policy, the applied norms during the implementation of LSSR are that all people are required to implement 3 M health protocol (e.g. wearing masks, washing hands with soap, and maintaining distance) and 3T (e.g. testing, tracking, and treatment) for medical personnel, government, and other institutions in accordance with the Regulation of the Minister of Health of the Republic of Indonesia Number 9 of 2020 concerning LSSR Guidelines. In a year, there has been a change in policy along with the increase in positive confirmed cases that eventually implement the newest 5 M health protocol namely wearing masks, washing hands with soap, maintaining distance, staying away from crowds, and limiting mobilization and interaction [18,19,22,23].

This study was conducted during the implementation of LSSR to CARE in Gresik, from May 2020 to January 2021. It was observed that there were no significant changes related to awareness of disaster response in implementing health protocols. This was indicated by the red status of the Covid-19 distribution zone. In addition, several violations were found such as not using a mask when doing activities outdoor, ignoring social distancing, and crowding in public places. There, moreover, were still parties who did not believe in the existence of Covid-19 and not few of them thought that this outbreak was God's destiny.

On the other hand, educating society has been carried out by local governments through task forces in each region starting from the small scale (e.g. RT/RW). The education is conducted through social and electronic media. A joint operation between Indonesian Army and National Police aims to enforce health protocols and limit community activities. However, in reality, violations are still existing. This is interesting to analyze regarding the construction built by the community as a conscious effort to respond to disasters in the midst of a pandemic and the dominant factors that affect the Covid Task Force in educating and enforcing rules during LSSR to CARE in Gresik.

Research related to people's responses to Covid has already been carried out. Rahmanti examines the public responses to the Government's policies on handling Covid [29]. The study shows that the trend of public sentiment is gradually shifting from “negative” to “positive” due to the government alertness and the spread of the disease which is starting to be controlled. The announcement of the Government's policy regarding the “new normal” issue received a positive response from the majority of Twitter social media users. Hamdi explained in his research that when the Indonesian Government closed mosques and large gatherings, the *Tabligh Jamaat* (Islamic Organization) saw Covid-19 as not a very dangerous thing [30]. They considered Covid-19 to be an anti-Islamic conspiracy. So they carry out the same worship activities as before Covid, namely gathering and preaching. Existing research focuses more on government policies and societal responses. Research that examines people's self-awareness of the dangers of Covid has not been studied. It is important to this study to understand how self-awareness results in a person's attitudes and actions in complying with health protocols and adherence to strict regulation.

This research is limited to awareness of community disaster response (Covid in the view of the Gresik community, awareness of complying with health protocols, awareness of complying with activity restriction policies) during the implementation of LSSR and CARE.

The obtained data were analyzed using Social Construction Theory of Peter L. Berger and Thomas Luckman. This theory explains that the analysis of social reality is built in two ways namely redefining between reality and knowledge and seeing society as an objective and subjective reality. In other words, individual is the shaper of society and society is also the shaper of the individual. In addition, this theory also explains a dialectical process that includes three simultaneous moments, namely externalization (adjustment to the socio-cultural world as a human product), objectification (interaction with the intersubjective world that is institutionalized), and internalization (individuals identified with institutions). Social organization of which the individual is a member is used to form a construct of reality [31].

## 2 Methods

This study used a descriptive qualitative research method to describe the object of research in the form of written or spoken words from people and observed behavior [32]. The focus of this study was the construction built by the community as a conscious effort to respond to disasters in the midst of a pandemic as well as analyzing the dominant factors affected the Covid Task Force in educating and enforcing rules during LSSR to CARE in Gresik.

The subjects of this study were those who were affected by the implementation of LSSR to CARE. Eight informants were selected through snowball sampling technique, whose social backgrounds were varied encompassing educators, community leaders, religious leaders, medical personnel, and general public. This study was carried out in Gresik during the implementation of PSSB until CARE, starting from May 2020 to January 2021. The reason for choosing the area was not only based on the risk of spreading Covid-19 in Gresik, but there were also violations related to social distancing policy such as recitations around the village, crowding in public places, holding wedding receptions exceeding the stipulated invitation quota, doing activities outside the home without wearing a mask, and vacationing in high-risk areas.

Data were collected by using in-depth interviews, questionnaires, and observations. The informants had understood the purpose of the study and had asked for confidentiality. In qualitative study, there was modeling that started from the process of categorizing the data sequence and organizing the data into one pattern, category, and large description unit, which further gave a significant meaning to the analysis. It also included explaining the pattern of description and looking for relationships between the dimensions of the description [32].

Data analysis and interpretation were carried out with a deep understanding of community construction as a form of awareness in understanding social reality. The construction included the emergence of the Covid Task Force (Satgas), society versus health protocol, the dialectic of health protocols in Gresik to build public awareness in the midst of a pandemic. Those three aspects of the community construction was referred to the dominant factors affecting the Covid Task Force in educating and enforcing rules during the implementation of LSSR to CARE in Gresik.

## 3 Results and discussion

Community disaster response awareness in the midst of the implementation of LSSR to CARE could be analyzed through construction built on knowledge and reality about disaster response and the spread of Covid-19. Based on the formation of the construction, it allowed the public to raise awareness in accordance with the thing being constructed, as well as the impact that appeared on the awareness that the community had made in dealing with the pandemic. The informants used in this study came from different socio-cultural backgrounds and were proven by their social status in society. So that, the construction was built from the community as the shaper of individual and the individual as the shaper of community [31].

### 3.1 Covid construction until the emergence of Covid Task Force (*Satgas*)

The construction of Covid in this study began with community's knowledge of disaster response including 1) believing as a preventive measure when a disaster occurred, 2) the government's response in preventing disasters, 3) activities carried out by the community individually and in groups as a form of sensitivity and conscious effort when a disaster occurred or as an anticipation of the unexpected impact, and 4) did not really understand the disaster response in detail.

Based on the knowledge of disaster response, it could be seen that there were two construction groups related to disaster response, namely knowing disaster response and not knowing disaster response. Groups who had good knowledge of disaster response could understand disaster response as a form of sensitivity, conscious effort, and active participation of the community as individuals and groups in dealing with disasters that occurred.

“… disaster response is a quick response carried out by a person as a conscious and sensitive effort to the environment when a disaster occurred and plays an active role in overcoming it” (FE Interview, 35 year old civil servant)

Meanwhile, the group who did not know about disaster response understood that disaster response was not important to know. During the Covid-19 pandemic, this group was more interested in information related to the economy than information related to norms that applied during the pandemic.

“I don't know what disaster response is and I don't want to know either. For me the information is not important” (Interview RN, 46 years)

“… rather than thinking about disaster response, I think how I can find money to feed my wife and children tomorrow. Problems like that should be left to the government, I don't want to get involved” (Interview RN, 46 years old)

The construction formed related to disaster response affected the community's construction of the current Covid-19 outbreak. This was due to the fact that handling during the pandemic included preparedness that must be carried out by all elements of society. In fact, the constructions built by the community related to Covid-19 were based on information obtained, habits, and the environment.

Community's construction based on the information obtained showed that people who updated information about Covid-19 had a high level of vigilance compared to those who were less updated. This could be proven from the questionnaires distributed in the community showing that around 45 % of the informants admitted to updating information on Covid-19, 20 % did not update the information very much, and the rest 35 % admitted that they did not update the information.

Informants who chose to update Covid-19 information thought that this disease outbreak was dangerous for human safety and health, so it was important to monitor developments starting from the number of confirmed cases, the symptoms caused, the distribution in their area, and the rules implemented during the outbreak. Such conditions were depicted in the following excerpts from interviews with the informant.

“I realize this virus is dangerous and deadly for patients who have heart disease, hypertension, diabetes, and lungs. Therefore, prevention is better for myself and my family. The way is to always update information both in online and electronic media” (FE Interview, 35 years old)

Informants who chose not to update Covid-19 information thought that simply knowing Covid-19 information was a better action so that they were not too afraid of the situation. This reality was reflected in the following interview excerpt.

“I know enough about the dangers and symptoms of Covid-19, I think it's enough to make things a little calmer and less prone to HOAX" (Interview EP, 23 years)

Informants who chose not to update information related to Covid-19 assumed that Covid-19 was a God's destiny and reminder for all humans, so that Covid-19 information became less important to know. They got focused on the economic life of the family as the impact felt during the pandemic. This reality was reflected in the following excerpts from the interview answers.

“Covid is a disease made by God, for example being exposed means it is God's destiny, what can't be done. Worried about not making the kitchen smoke every day” (Interview RN, 36 years).

“… Death is God's destiny not because of Corona, until now I am still out and still working as usual. Because for me the more we are alert, the effect becomes fear etc.” (IP Interview, 26 years old)

The construction that the community understood related to Covid-19 actually confirmed Berger's idea that conveyed that a construction built from knowledge became a picture of reality occurred and individual knowledge was seen as a picture of objective reality in itself [33]. This knowledge was reflected when individuals constructed Covid-19 as an objective reality.

Based on the construction differences existed in the community and the addition of confirmed cases that continued to occur, the government had formed a task force for educating the public regarding the prevention and control of Covid-19 through health protocols and handling the spread of Covid-19. Government policies starting from the implementation of 3 M, 3 T, and 5 M were a form of norms and culture that must be instilled during the pandemic.

Further, there would be no misunderstanding of the information received and obtained by the public. The Task Force (*Satgas*) served as the second guard after health workers and was formed on a macro (national) to micro (RT/RW) scales. At the micro level, the Task Force was responsible to establish a Covid post, conduct raids (sweeping) targeting mass crowds in village areas, coordinate with health workers if there were confirmed cases, and sterilize public places (e.g. schools, places of worship) [26]. This reality was revealed from the statement of the Covid Task Force in the following village.

“Our duties start from providing a place for isolation for residents from outside the city, providing hand sanitizer when entering the village, checking ID cards, establishing a one-door system, and not tolerating residents who do not wear masks, if they want to go out they must wear masks. Not wearing a mask is prohibited from leaving or entering the village. All of this is an effort that we are doing to prevent the spread of Covid-19” (MU Interview, 39 years old)

Active community participation was an important element after good cooperation between the community and the Task Force in preventing the spread of Covid-19 through implementing health protocols. In fact, this reality was reflected in the expression of one of the informants.

“Without the active role of individuals and groups, this Covid-19 will not end. Therefore, all parties must be willing to work together so that the pandemic ends quickly” (AZ Interview, 25-year-old Medical Personnel)

## 4 Society and health protocol

The implementation of health protocols in the community rose various problems. On the one hand, they must implement health protocols starting from wearing masks, washing hands with soap, maintaining distance, staying away from crowds, and limiting mobility and interaction. However, this situation created uncertainty, confusion, and shock as a result of the emergency spread of Covid-19 [34].

### 4.1 Wearing a mask

The first health protocol focused on wearing a mask. The reason for wearing mask was in accordance with the results of research conducted by experts in the world regarding the rapid spread of Covid through air [[35], [36], [37]]. The reality regarding the use of masks showed that people had not fully accepted the new habit because it was contrary to the culture of the people who were not used to doing activities by wearing masks.

Sociologically, this happened because the habitus that went on in their social life did not claimed that using masks was part of the efforts to maintain health. So, when there was a sudden change in the events of the Covid-19 pandemic, it created chaos and culture shock in the community because they were not used to it and there were no culturally binding rules to use it. However, on the other hand, they had the obligation to use it as an effort to prevent and spread Covid-19.

Cultural shock could be seen from the response of the community who considered that the new habit was contrary to the habit in their social environment and was reflected in the informant here.

“Honestly, I'm also one of the people who often don't use masks, because I'm not used to wearing masks, its uncomfortable when I breathe and my friends also feel the same as me. After all, this is destiny and God's decree, so just live it” (Interview RN, 46 years old)

This statement was confirmed by the Covid-19 Task Force in the village.

“… it is true, one of the reasons why many people do not wear masks is because they are not used to wearing masks. Before this Corona, people could be said to have never used a mask, so seeing the government's call to wear a mask when leaving the house, we are trying to reinforce the rules" (MU Interview, 39 years old)

This showed that people's knowledge depended on the culture developed by the surrounding environment. According to Berger, this condition was an intersubjective experience referred to the dimensions of the structure of general awareness towards individual awareness in the midst of integrated and interactive social situations [31]. This was because individuals in society had become an inseparable part and their reactions were part of response to what they thought and the surrounding environment.

### 4.2 Washing hands

Washing hands was not a new thing in Gresik's social environment. This was due to the fact that the people had a culture of washing their hands as a form of cultural ritual when visiting religious tourism sites, ablution, and rituals before and after eating. However, in reality, there is a gap between the existing culture and new habits in the midst of a pandemic.

Several informants in this study stated that such conditions were recognized as differences. The difference lied in the knowledge built on the habit of washing hands. Washing hands in the middle of a pandemic was understood as an ordinary ritual, not something new, so people had a tendency to ignore the warning to wash hands. Meanwhile, washing hands was carried out as a form of cultural ritual when visiting religious graves. This was reflected in the statement of the informants.

“Washing hands in religious places has a magical value compared to the recommendation to wash hands when pandemic. Maybe because of that, people find it difficult to apply the habit of washing their hands properly the recommendation of the Covid Task Force” (IP Interview, 26 years old)

This statement was confirmed by the Covid Task Force who stated that there was a different perspective between the culture practiced by the community for a long time and the new habits to prevent the spread of Covid-19 in Gresik.

“I admit that it is difficult to raise public awareness to do things out of the ordinary, but seeing conditions like this it must and must be done” (EP Interview, 23 years old)

“Covid is a real disease, it can be prevented. If everything is related to Fate, it cannot be used as a basis for not implementing health protocols. Even though it's true that everything that happens depends on God's destiny” (MU Interview, 39 years old)

This reality showed that knowledge about the importance of public awareness to obey the rules applied during the pandemic was still not implemented properly. In this case, the role of the Covid Task Force was to regulate changes in values and norms that applied in society due to the pandemic. The goal was to raise public awareness in obeying the rules.

In addition, the reality formed in social construction showed the existence of individual cognitive work to interpret the world of reality existed as a result of social relations between individuals and their surrounding environment. In addition, individuals built their own knowledge of the reality they saw based on pre-existing knowledge structures. In this case, humans were said to be social beings, therefore, every statement must be proven true and the key to true knowledge was facts [38]. For this reason, reality and knowledge could not be separated in analyzing social construction problems in life.

### 4.3 Social culture

Social culture referred to health protocols covering maintaining distance, staying away from crowds, and limiting mobilization and interaction. The term *social culture* was chosen to represent the health protocol, while at the same time showing that the socio-cultural community could not be separated from the protocols to support the Task Force to prevent the spread of Covid-19 in Gresik.

From May 2020 to January 2021, Gresik government had implemented Large-Scale Social Restrictions (LSSR) and the Community Activity Restrictions Enforcement (CARE). Unfortunately, violations still happened with common reasons such as ignorance, inadvertence, and discomfort, especially for the case of social distancing, avoiding crowds, limiting mobilization and interaction. For those who did violations, the condition was not one of the cultures in their daily lives. Many of them, for instance, gathered in coffee shops, cafes, and other crowded places.

This was confirmed by the Task Force when accompanying judicial operations with Indonesian National Army -Indonesian Republic Police, Transportation Agency, Civil Service Police Unit, and medical officers at a coffee shop.

“… there were 46 violators, namely crowding in coffee shops and not wearing masks. 15 people received push-up sanctions, 17 verbal reprimands, and 14 coffee shop visitors were carried out quickly and the results were two reactive people. This is the impact if you don't comply with health protocols” (Interview AZ, health worker, 25 years old).

In addition, there were still some recitations that did not apply social distancing. There were also people who held weddings that exceeded the invitation quota. This reality was obtained based on field and media observations as well as the interview results.

“… it makes no difference whether or not there is Corona. There are still a lot of coffee shops that are open and still busy, don't wear masks, there are still people at the recitation, there's no social distancing …”

“Some time ago I also received information from my neighbor, he said that at his friend's residence there were still those who were desperate to hold a wedding reception. Even though the Lurah (Headman), RT/RW (Neighborhood Association/Citizens Association) was informed, but they were still desperate. Finally the police disbanded” (Interview IP, 26 years old).

Such conditions indicated that the community was adjusting to new rules. For Berger, this process was classified as an externalization construction process, meaning that society as subjective and objective stakeholder had a role to adapt to the socio-cultural aspect as a human product. The adjustment in relation to efforts to prevent the Covid-19 pandemic was conducted by implementing wearing masks, washing hands with soap, maintaining distance, staying away from crowds, and limiting mobilization and interaction during LSSR to CARE.

Humans made such rules in an effort to realize social order. The existing violations were considered as the inability of the community to face and adapt to the rules used to maintain social order. Therefore, the problem of change was in the process of externalization. People who used to being in order did not mind the social changes occurred, but those on the contrary still made deviations to adjust to their social roles. In other words, violations continued to occur.

In fact, the informant RN also experienced difficulties in adjusting to the rules.

“..My life is already difficult, the Covid-19 has made it more difficult for my economy and made it difficult for me to adjust to the applicable regulations. From those who are not used to wearing masks to wearing masks, those who are not used to keeping their distance, not crowding, and not being used to doing activities at home, all are forced to be followed. Until I thought, is this disaster a warning from God? God's destiny really tests patience” (interview RN, 46 years)

## 5 Dialectic of health protocol in Gresik

There were three dialectical components in Peter L. Berger's social construction namely internalization, objectification, and externalization. The dialectic of health protocols was reflected as follows.

a. Internalization

The internalization process that emerged as a form of self-identification with socio-cultural was originated from public knowledge about Covid and disaster response efforts manifested in the active participation to assist medical personnel and the government in overcoming Covid-19 by complying and implementing health protocols. For Berger, this process contained the construction of knowledge as a picture of the reality and individual knowledge was seen as a picture of the objective reality [33]. This knowledge was reflected when individuals constructed Covid-19 as an objective reality.

b. Objectification

The objectification process that emerged as an awareness made to other parties (Covid Task Force) and adjustment to the rules applied in society was realized through self-interaction with socio-cultural aspect to face Covid-19 pandemic. This process included changes in habitus or habits carried out by the community. Before this outbreak, people did not have strict rules regarding wearing masks when leaving the house, coughing and sneezing etiquette according to the rules, carrying out a clean and healthy lifestyle, and washing hands regularly. However, after this outbreak emerged, people faced a situation that changed their habituation by applying what they were not used to doing.

c. Externalization

The process of externalization appeared as a form of adjustment to the socio-cultural aspect as a human product. This was realized through adjustments to the current rules such as conducting 5 M health protocol to prevent the spread of Covid-19.

Through this dialectical process, community's disaster response awareness in dealing with the Covid-19 pandemic could be initiated such as by wearing masks, maintaining social distance, washing hands according to suggested procedures, and applying good coughing and sneezing etiquette. All these processes were parts of the objective and subjective dimensions, because society was a cultural product of society itself (the existence of inter subjective relationships) and human existence as well as the creator of their own world be [31].

Based on the findings and the dialectical process occurred, it was found that the dominant factor that influenced the Covid Task Force in educating and enforcing the rules during the LSSR to CARE in Gresik was the strong socio-cultural factors adopted by most people. They thought that this outbreak emerged as a reminder, warning, and destiny from God. So that, the efforts of the Covid Task Force to carry out education (e.g. wearing masks, washing hands, etc.) and policies related to preventing the spread of the virus were hampered by these factors.

Thus, the relationship between individuals and their institutions was a dialectic (intersubjective) expressed in three moments: society as a human product; society as an objective reality, and human as a social product. This dialectic was mediated by experience and roles that represented individuals in an institution [39].

Referring to the dialectic, the COVID-19 pandemic could not be separated from the theological debate about destiny. The theme of destiny gave dissimilarities to a person's behavior in responding to the pandemic and had an impact on compliance with health protocols. A person who ignored health protocols during a pandemic was motivated by the theological belief that God had arranged life and death, including the presence of Covid. As a result, a person thought that they did not need to wear a mask, wash hands, and keep a distance, and so on. Social and religious activities presented by many people without heeding the health protocols were still ongoing. The reason was the belief that efforts to comply with health protocols were useless if in the end God determined someone's destiny.

Such society's conception, if traced, led to the understanding of destiny in the perspective of theology. The *Jabbariyah* perspective held that the will or action was carried out by force (fatalism) because God had determined their destiny. Humans were passive and did not have the ability to change their destiny. Consequently, the Covid-19 pandemic was believed to be a disease made by God, a warning from God. A person would be exposed to Covid when God determined so, even though he had strictly adhered to health protocols. Similarly, a person would die when God predestined. The impact of this understanding was the neglect of the government's regulations.

In regard to the *Qadariyah* perspective, they relied on human ability to deal with problems without God's intervention. Humans were active so that the will absolutely depended on human free will and free act. In this context, the Covid-19 pandemic was understood as a factual reality that was dangerous and led to a human-made disaster. Consequently, individuals obeyed health protocols [40]. In addition to these two understandings, there was also a group of people who had a progressive theological understanding that prioritized preventing the occurrence of human dangers over other benefits. This progressive theology was based on the principle of *Al-dharuriyah al-khamsah,* the goals of Islamic law covering preserving religion, soul, reason, offspring, and property. Covid 19 threatened human life, therefore, the benefit of the soul must be prioritized.

The influence of *Jabbariyah* ideology encouraged counterproductive behavior towards efforts to cheese the spread of the corona virus. While the influence of Qadariyah's understanding was enough to help prevent the further spread of the Corona virus. Meanwhile, the influence of progressive Islamic theology encouraged productive behavior by being actively involved in carrying out social transformation in efforts to prevent and control Covid-19 [41].

From a historical perspective, pandemics had occurred among Muslims in the era of Prophet Muhammad. The Prophet's pandemic prevention and control was explained in the hadith “When you hear of a plague in an area, then don't enter it. But if a plague occurs in the area you are in, then do not leave that place.” [42]. This hadith was relevant to Covid-19 mitigation, such as lockdown, self-quarantine, self-isolation, staying at home, maintaining distance, Large-Scale Social Restrictions (LSSR), and Enforcement of Community Activity Restrictions Enforcement (CARE).

The discourse on the Covid-19 pandemic associated with awareness of disaster response ultimately narrowed how an individual was able to have a dialogue between himself and the sociocultural aspect, as well as a dialogue between his theological conceptions and the social reality faced. Individuals were in objective and subjective reality. During the pandemic, there was a set of rules that served as guidelines for preserving social order for the sustainability of human life. However, even though the rules in the social structure were restrictive, there were many violation of health protocols. Violations of these rules were caused by inconsistent processes of individual externalization or individuals’ incapacitation to apply the rules to maintain social order.

## 6 Conclusion

As a conclusion, first, socially, the community has not taken the dangers of Covid-19 seriously, which is proven by violations of the rules when implementing LSSR and CARE. For instance, people have not implemented 5 M properly. Second, there is an inconsistency between knowledge and social reality related to disaster response as the first step to construct something important in everyday life. On the other hand, many people are aware of the dangers of Covid-19, but they have not shown good awareness. Third, the dialectical process of social construction theory by Peter L. Berger and Thomas Luckman shows that the community has not implemented a disaster-aware culture by obeying the rules as a socio-cultural product of the inter subjectivity. Fourth, the socio-cultural environment determines a person's construction to carry out self-identification, self-interaction, and adjustment to social changes that occur in daily life, especially in the face of the Covid-19 pandemic. Its relation in shaping individual and collective consciousness has hampered the responsibilities carried out by the Task Force. It is proven that during a year of the spread of Covid, Gresik still becomes the top 10 areas with the highest confirmed Covid-19 cases in East Java Province.

In the context of the Gresik community, the religious approach is important as an instrument for forming self-awareness. This religious approach involves involving religious leaders as a model for complying with health protocols and government regulations.

Based on previous findings by Ref. [43], actions that can be taken to reduce the spread of COVID-19 are campaigning for public awareness, testing policies, and maintaining social distancing. in addition, according to Ref. [44], pharmaceutical and non-pharmaceutical measures also have a great impact in reducing the spread of Covid-19 such as restrictions on community mobility, financial assistance for poor families, providing free vaccines and vitamins.

The novelty of this research is the finding that theological elements play a role in controlling Covid-19, this finding requires the importance of government policies in disaster management with various approaches, namely social, cultural, health, security, politics and also religion. Based on the results of this study, it is necessary to conduct further research related to the model of improving people's healthy lifestyles with multiple approaches. In the early stages, the research focused on the theological approach.

## Ethics statement

This study was reviewed and approved by Institution of Research and Community, Universitas Negeri Surabaya, with ethics approval reference [Number: 108065/UN38.III.I/TU.February 00, 2023].

## Data availability statement

All the raw sequence data in this study are available at https://ourworldindata.org/covid-cases or https://www.kompas.com/tren/read/2021/03/15/095100365/update-corona-dunia-15-maret--120-juta-kasus-covid-19-10-negara-dengan?page=all for Table 1 and available at https://infeksiemerging.kemkes.go.id/situasi-infeksi-emerging/situasi-terkini-perkembangan-coronavirus-disease-covid-19-15-maret-2021 for Table 2.

## Funding

This research received no external funding.

## CRediT authorship contribution statement

**Sarmini:** Writing – review & editing, Writing – original draft, Visualization, Validation, Methodology, Conceptualization. **Faridatul Lailiyah:** Writing – review & editing, Visualization, Resources, Methodology, Formal analysis, Data curation. **Suprapto:** Writing – review & editing, Writing – original draft, Visualization, Supervision, Resources, Methodology, Conceptualization. **Mutimmatul Faidah:** Writing – review & editing, Writing – original draft, Resources, Data curation, Conceptualization.

## Declaration of competing interest

The authors declare the following financial interests/personal relationships which may be considered as potential competing interests:Sarmini Sarmini reports administrative support, article publishing charges, and writing assistance were provided by State 10.13039/501100012696University of Surabaya. Sarmini Sarmini reports a relationship with State 10.13039/501100012696University of Surabaya that includes: employment and non-financial support. Sarmini has patent issued to State University of Surabaya/Universitas Negeri Surabaya. The Author Declare, there is no conflict of interest If there are other authors, they declare that they have no known competing financial interests or personal relationships that could have appeared to influence the work reported in this paper.
